# Dynamics of Cattle Production in Brazil

**DOI:** 10.1371/journal.pone.0147138

**Published:** 2016-01-27

**Authors:** Concepta McManus, Júlio Otávio Jardim Barcellos, Bruna Krummenauer Formenton, Potira Meirelles Hermuche, Osmar Abílio de Carvalho, RenatoFontes Guimarães, Miguelangelo Gianezini, Eduardo Antunes Dias, Vinícius do Nascimento Lampert, Daniele Zago, José Braccini Neto

**Affiliations:** 1 INCT-Pecuária, Universidade de Brasília, Brasília, DF, Brasil; 2 NESPRO, Departamento de Zootecnia, Universidade Federal do Rio Grande do Sul (UFRGS), Porto Alegre, Brasil; 3 Universidade de Brasília, Brasília, DF, Brasil; 4 Empresa Brasileira de Pesquisa Agropecuária, Bagé, RS—EMBRAPA-CPPSUL, Brasil; Federal University of Parana (UFPR) ) – Campus Palotina, BRAZIL

## Abstract

Movement of livestock production within a country or region has implications for genetics, adaptation, well-being, nutrition, and production logistics, particularly in continental-sized countries, such as Brazil. Cattle production in Brazil from 1977 to 2011 was spatialized, and the annual midpoint of production was calculated. Changes in the relative production and acceleration of production were calculated and spatialized using ARCGIS®. Cluster and canonical discriminant analyses were performed to further highlight differences between regions in terms of cattle production. The mean production point has moved from the Center of Minas Gerais State (in the southeast region) to the North of Goiás State (in the Midwest region). This reflects changes in environmental factors, such as pasture type, temperature and humidity. Acceleration in production in the northern region of Brazil has remained strong over the years. More recently, “traditional” cattle-rearing regions, such as the south and southeast, showed a reduction in growth rates as well as a reduction in herd size or internal migration over the period studied. These maps showed that this movement tends to be gradual, with few regions showing high acceleration or deceleration rates.

## Introduction

The occupation of geographic space and territory has been a constant concern of agribusiness and society and, consequently, of public policies. However, beef demand is growing worldwide [1] and stimulates increased productivity and production. This has occurred mainly in countries with areas for expansion of beef cattle, such as Brazil. Only between 1990 and 2009, the Brazilian cattle herd has grown by 33%, mainly through expansion in the Midwest region, due to the presence of several favorable elements, such as the presence of external drivers, including socioeconomic factors [2].

However, livestock development within a country or region has implications on several fronts (genetics, adaptation, well-being, nutrition), particularly in continental-sized countries such as Brazil. This movement in production is also accompanied by the need for adequate infrastructure (abattoirs, transport, energy, inputs industries), commercialization and marketing, as well as technical support. It also fuels the need for credit programs as well as research and development activities to support intensification goals [3].

Furthermore, statistical analyses have shown only quantitative changes in herd productivity indicators and associated regions and do not enable a more detailed temporal interpretation to understand the different phenomena and dynamic trends of an activity in the territory. Thus, the use of Geographical Information Systems (GIS) has resulted in a better and more timely visualization of production systems and can aid in the identification of problematic areas [4]. GIS can aid in the identification of areas that are suitable for the expansion of production [5], as well as identification of genetic resources for conservation [6]. Thus, the objective of this article is to study the dynamics of cattle production in Brazil on a municipal scale and to identify potential areas of strangulation for continued growth and variables that could limit the increase in production and productivity of the cattle in Brazil.

## Methods

This study considered all municipalities (5561 in total) in five regions of Brazil. Data on cattle production in Brazil were obtained from the website of the Brazilian Institute for Geography and Statistics [7] from 1977 to 2011.

Maps were generated using the number of animals/head of cattle (production) for each year, which was then converted into a raster format (GRID) and processed using ENVI 4.5 software.To analyze the dynamics of production growth, maps were drawn for the relative growth rate (GR %) of production by municipality by dividing the time interval analyzed into seven equal periods, using the sum of 5 years of production in each period examined (1977 to 1981 1982 to 1986, 1987 to 1991, 1992 to 1996, 1997 to 2001, 2002 to 2006, and 2007 to 2011). The relative growth rate was calculated using the following equation (Eq 1):

$$\text{GR}=\frac{\left[(\text{Production in present period-Production in previous period})\times 100\right]}{\text{Production in previous period}}$$ Eq 1

The relative growth rate resulted in seven maps. Data were classified as high growth (growth ≥ 50%), low growth (between 10 and 49.9%), stagnation (between +9.9 and -9.9%), low reduction (between -10 and– 49.9%) and high reduction (≤ -50%). The acceleration of growth rate was obtained by the relative differences in the images, which resulted in six maps calculated as GRPsP − GRPvP, where GRPsP is the Growth Rate in the Present Period and GRPvP is the Growth Rate in the Previous Period. These maps were generated on a continuous scale, as these represent differences between two periods, presented as the beginning of one period until the end of the next period (p.ex 1977 to 1986).

The spatial midpoint of production in the country was calculated for each year to assess the direction of production in the country. The latitude (Eq 2) and longitude (Eq 3) midpoint were obtained by multiplying the sum of the geographic coordinates of the municipality and their production divided by the number of municipalities for each year.

$$\frac{\sum \left(\mathrm{latitude}\times \mathrm{production}\right)}{\mathrm{Number of municipalities}}$$ Eq 2

$$\frac{\sum \left(\text{longitude}\times \text{production}\right)}{\text{Number of municipalities}}$$ Eq 3

Cluster (PROC CLUSTER) and canonical discriminant (PROC CANDISC) analyses were performed to further highlight differences between regions in terms of cattle production using SAS® v.9.3 (SAS Institute, Cary, North Carolina). Two groups of clusters of municipalities were formed according to the relative growth and acceleration calculated above. Cluster means for acceleration and relative growth were compared using an analysis of variance and mean separation using Tukey test (P<0.05) after standardization. Discriminant canonical analyses were used to identify regions and municipalities that behaved in a different manner.

## Results

Several different movements have been observed over the years: North (1977–1991; 2003–2011) Midwest (1978–1981; 1991–1995) and Northwest (1995–2003). Cattle numbers grew in all regions over the period studied (Table 1). The most expressive growth was initially observed in the Northeast and Midwest regions, which sustained this growth over the period; however, this growth has been slower in recent years (Table 2).

**Table 1:** Relative growth of cattle numbers and percentage (standard error) in Brazil from 1977 to 2011 in 5-year periods by region.

	Year Interval													
Regions	77–81		82–86		87–91		92–96		97–01		02/06		07/11	
	N°head	%	N°head	%	N°head	%	N°head	%	N°head	%	N°head	%	N°head	%
MW	298,900a	43.31b	388,884a	17.13b	969,797a	25.22a	564,002a	14.58b	621,752a	14.45b	749,685a	8.83c	757,887a	9.04b
	(5582)	(7.91)	(5821)	(3.24)	(6156)	(4.75)	(6833)	(3.48)	(7076)	(1.51)	(8470)	(1.54)	(8362)	(0.8)
N	66,973cd	375.64a	101,116b	78.58a	144,380b	27.91a	196,092b	32.84a	255,299b	43.67a	415,802b	39.83a	451,584b	19.1a
	(1691)	(146.53)	(2102)	(24.19)	(2901)	(5.89)	(3026)	(7.71)	(3193)	(3.95)	(5766)	(4.48)	(6691)	(3.2)
NE	580,62d	30.41b	61,998c	13.38b	71,232d	8.59b	66,548d	-1.81c	63,395d	0.10c	72,338c	22.76b	80,417c	8.76b
	(442)	(1.45)	(452)	(1.25)	(465)	(0.77)	(475)	(1.22)	(465)	(2.05)	(492)	(1.18)	(549)	(1.44)
S	95,551bc	18.76b	103,429b	5.77b	106,362cd	-0.34c	109,898c	6.14bc	111,485c	1.62c	116,694c	2.79c	115,926c	9.53b
	(1421)	(0.96)	(1450)	(1.17)	(1421)	(0.90)	(1348)	(1.34)	(1220)	(0.95)	(1302)	(1.3)	(1262)	(0.94)
SE	106,246b	2.35c	105,098b	5.83b	108,424bc	5.23bc	111,719c	3.27c	110,930c	2.78c	116,476c	8.24c	115,183c	5.73b
	(772)	(2.91)	(816)	(1.42)	(864)	(0.84)	(819)	(0.90)	(765)	(1.29)	(822)	(0.77)	(807)	(0.89)
Average	229,658	94.09	152,105	24.13	280,039	13.32	209,652	11.00	232,572	12.52	294,199	16.49	304,1994	10.43
Total	1,148.29		760,525		1,400.20		1,048.26		1,162.86		1,470.995		1,520.997	

MW–Midwest; N–north; S–South, SE–Southeast, NE–Northeast. Means in the same column with different letters indicate a difference according to the Tukey test (P<0.05).

**Table 2:** Cattle production in Brazil from 1977 to 2011 in 5-year periods per region with regard to the number of head and percentage acceleration (standard error).

Regions	Year Interval													
	77–81		82–86		87–91		92–96		97–01		02/06		07/11	
	N°head^1^	%	N°head	%	N°head	%	N°head	%	N°head	%	N°head	%	Nºhead	%
MW	59,780a	-5.27a	77,777a	-31.3a	93,959a	-5.19b	112,800a	-14.35b	124,350a	-3.42a	149,937a	-7.62c	151,577a	0.22a
	(5582)	(7.70)	(5821)	(7.84)	(6156)	(3.11)	(6833)	(5.63)	(7076)	(3.67)	(8470)	(1.68)	(8362)	(1.60)
N	13,395b	352.82a	20,223b	-308.67b	28,876b	-52.94a	39,218b	-3.24ab	51,060b	2.46a	83,160b	-3.94c	90,317b	-20.69b
	(1691)	(146.71)	(2102)	(150)	(2901)	(24.90)	(3026)	(10.25)	(3193)	(8.63)	(5766)	(5.91)	(6691)	(5.52)
NE	11,612b	11.34a	12,400b	-16.95a	14,246c	-5.35b	13,310c	-10.86b	12,679c	1.00a	14,468c	22.52a	16,083c	-14.12b
	(442)	(2.06)	(452)	(2.05)	(475)	(1.54)	(465)	(1.66)	(378)	(1.69)	(492)	(1.66)	(549)	(1.63)
S	19,110b	6.60a	20,686b	-13.1a	21,272c	-5.8b	21,980c	5.19a	22,297c	-6.24a	23,339c	1.20b	23,185c	6.74a
	(1421)	(1.38)	(1450)	(1.59)	(1421)	(1.43)	(1348)	(1.75)	(1220)	(1.83)	(1302)	(1.47)	(1262)	(1.16)
SE	21,249b	-26.47a	21,020b	2.64a	21,685c	-0.68b	22,344c	-2.23ab	22,186c	-1.16a	23,295c	4.39b	151,577a	-2.45a
	(772)	(3.34)	(816)	(3.22)	(864)	(1.70)	(819)	(2.82)	(765)	(1.69)	(822)	(2.43)	(8362)	(1.88)
Average	25,029	67.80	30,421.20	-73.47	36,007.60	-13.99	41,930.40	-5.09	46,514.40	-1.47	58,839.80	3.31	86,547.80	-6.06
Total	125,146		152,106		180,038		209,652		232,572		294,199		432,739	

^1^ Number of animals per municipality per year; MW–Midwest; N–north; S–South, SE–Southeast, NE–Northeast.

Means in the same column with different letters indicate a difference according to the Tukey test (P<0.05).

**Fig 1:**
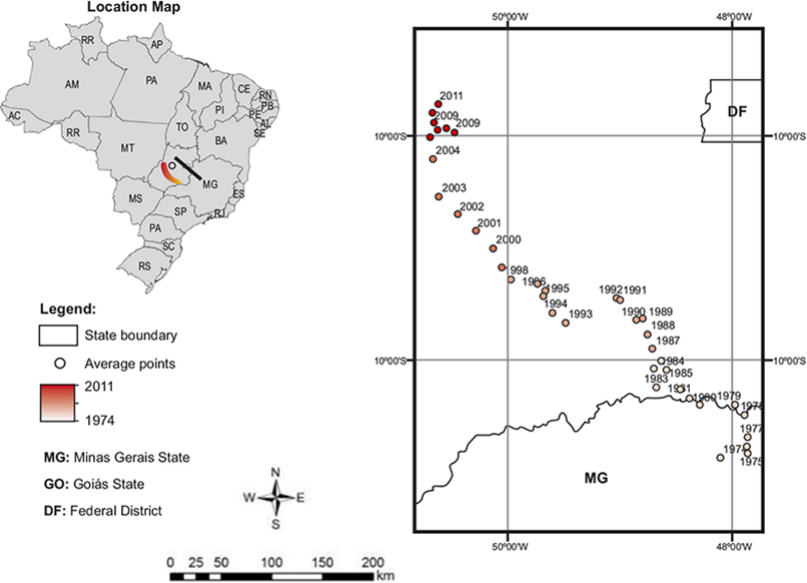
Midpoint of cattle production in Brazil by year.

This indicated that the mean production point has moved from the Center of Minas Gerais State (in the southeast region) to the North of Goiás State (in the Midwest region) (Fig 1). This change also reflected a strong change in environmental factors such as NDVI (Normalized Difference Vegetation Index), temperature and humidity. The northeastern region underwent a deceleration in the 1990s similar to the southeast at the end of this period. The southeastern and southern regions showed the slowest growth during these periods.

**Fig 2:**
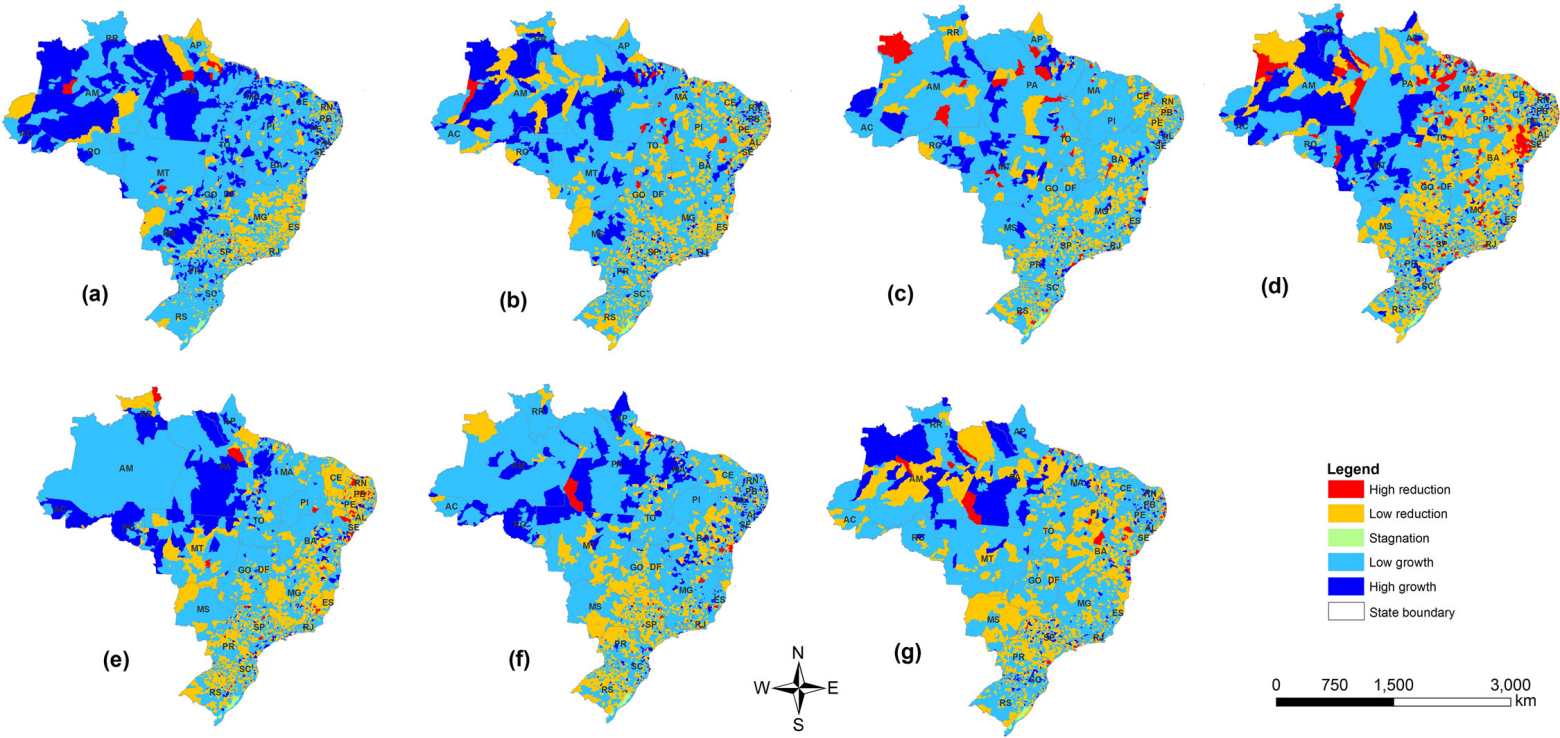
Growth of cattle production in Brazil by municipality by period: (A) 1977–1981; (B) 1982–1986; (C) 1987–1991; (D) 1992–1996; (E) 1997–2001; (F) 2002–2006; and (G) 2007–2011.

In recent years, strong growth was observed in the northern region of Brazil (Fig 2G) with a reduction in the south of the Midwest region and along the northern border with Colombia. This growth in the north can be observed from the early 2000s (Fig 2E–2G); however, this reduction is a more recent phenomenon. Growth in the Midwest was observed in earlier years (Fig 2A–2C). While the Pantanal region of Brazil showed strong growth in cattle numbers in the 1970s (Fig 2A), the growth has recently been reduced (Fig 2G).

**Fig 3:**
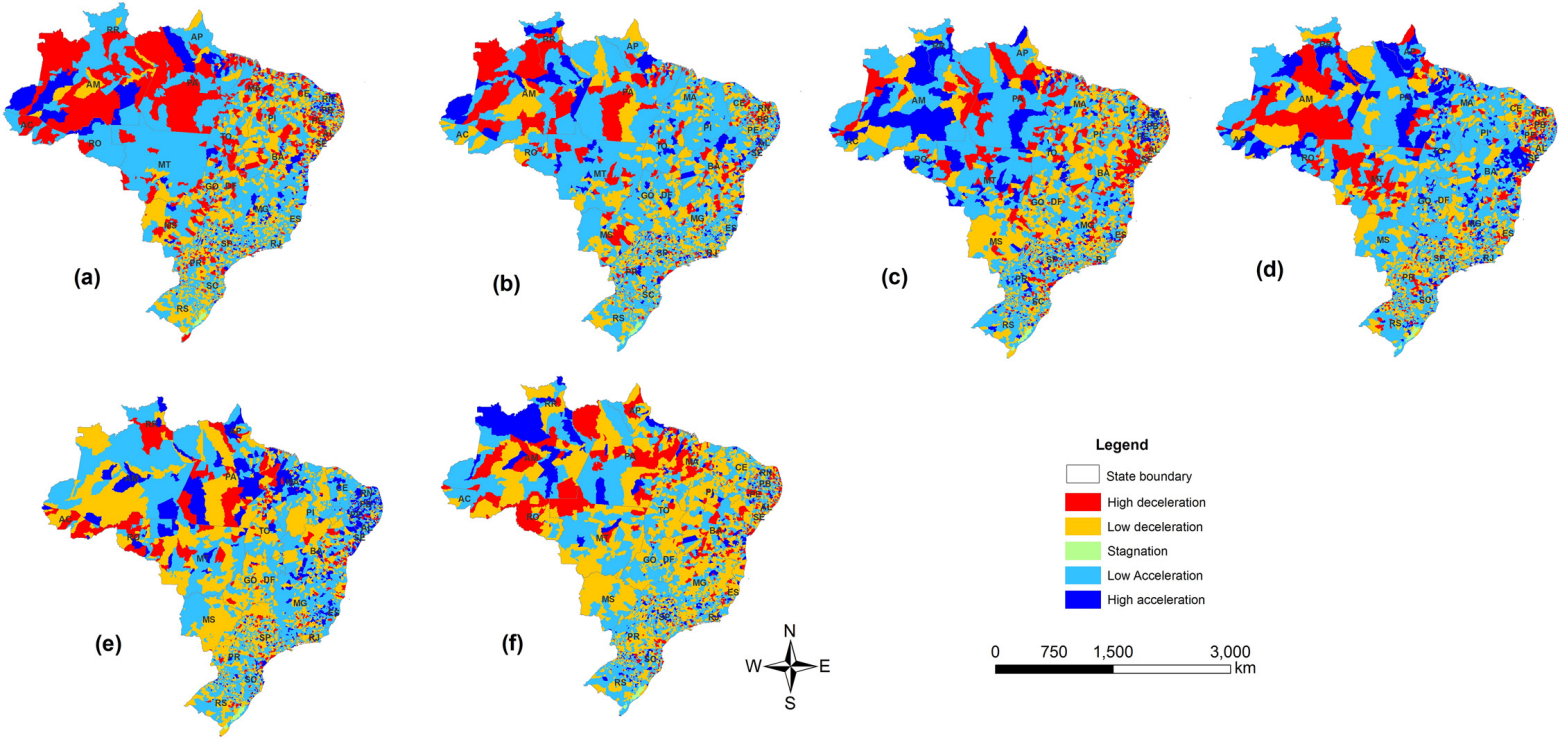
Acceleration of Cattle Production in Brazil by period: (A) 1977–1986; (B) 1982–1991; (C) 1987–1996; (D) 1992–2001; (E) 1997–2006; and (F) 2002–2011.

The acceleration in production in the northern region of Brazil has remained strong over the years (Fig 3), but the southern part of this region and Midwest showed stagnation in the early years of this study. More recently, these regions have shown a reduction in growth rates and “traditional” cattle-rearing regions, such as the south and southeast, which, in general, have demonstrated a reduction in growth rates as well as a reduction in herd size or internal migration over the period studied. The maps showed that this movement tends to be gradual, with few regions showing high acceleration or deceleration rates.

**Fig 4:**
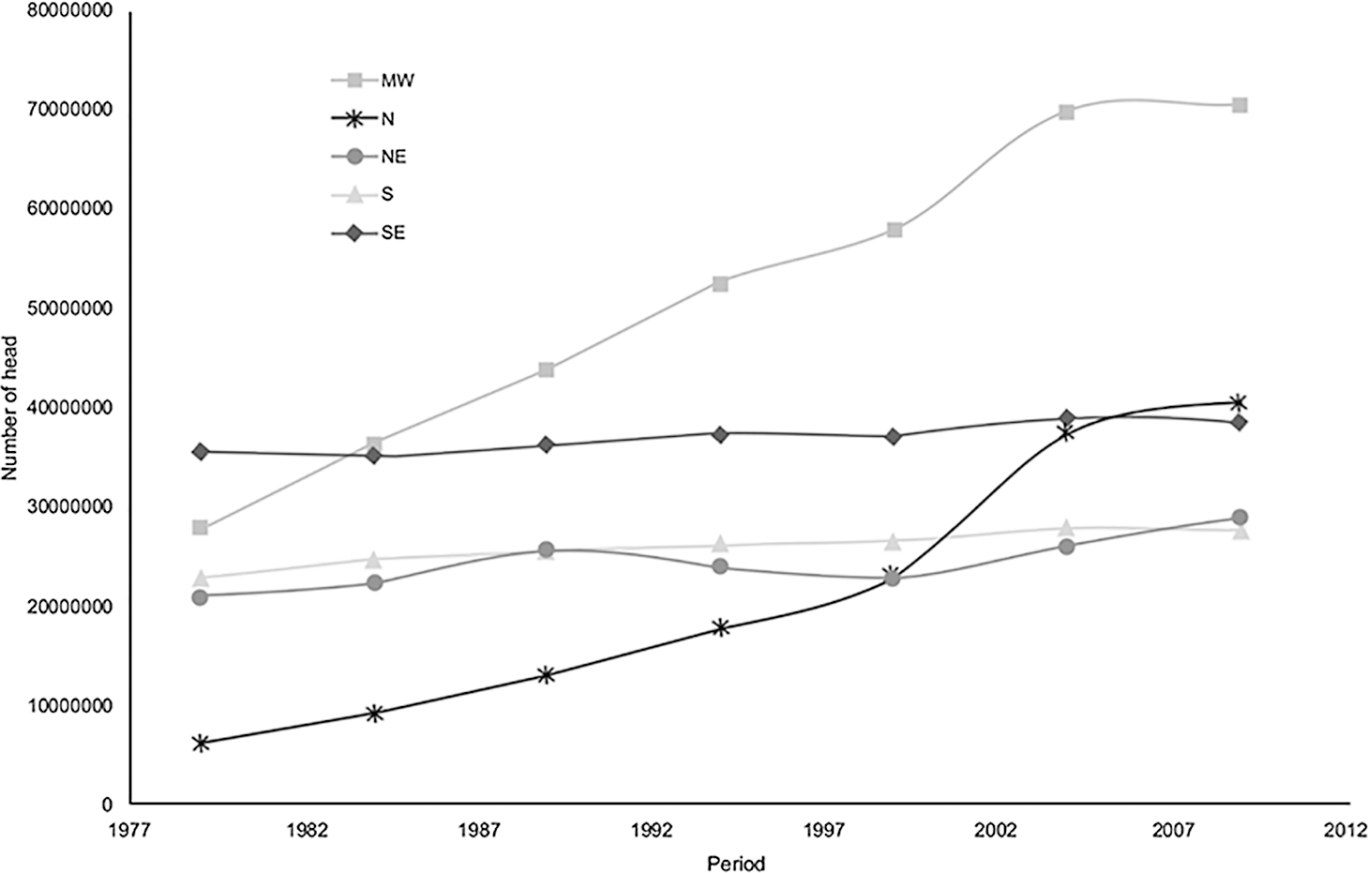
Cattle numbers by region in Brazil (1977–2012) with error bars.

The relative increase in production in the Midwest and northern regions is evident in Fig 4, which is considerably higher compared to other regions. In the northern region, because the original herd was small, the initial acceleration (1980–1990) was high with the migration of cattle production from other regions, but in numeric terms, the herd size has only become expressive within the last 10 years. Although there was a high acceleration in production in the region since the 1980s, this growth has decreased significantly in recent years.

The Midwest and northern regions are highlighted for their increase in cattle production over the period studied, in particular, the states of Mato Grosso do Sul, Goias, Mato Grosso (Midwest region) and Roraima (northern region). These two regions are also important for acceleration in production, which is specifically highlighted in the Northern states of Para, Roraima and Acre (Northern region) as well as the Midwestern state of Mato Grosso.

Clusters formed according to relative growth and acceleration data showed that a small part of the municipalities is responsible for the largest relative growth (Table 3, cluster 4) and acceleration in growth (Table 4, cluster 3). Importantly, these clusters do not indicate the same group of municipalities in the two analyses.

**Table 3:** Clusters of Brazilian municipalities according to the relative growth in the number of cattle (standard error) and the percentage of the total herd from 1977 to 2011 in 5-year periods.

Cluster	N° of municipalities	Cluster totals													
		77–81		82–86		87–91		92–96		97–01		02–06		07–11	
		N° head	%	N° head	%	N° head	%	N° head	%	N° head	%	N° head	%	N° head	%
1	4586	112,079,170^a^(781)	99.33^a^	12,339,5441^a^(830)	97.01^a^	13,363,0443^a^(886)	92.97^a^	13,531,0694^a^(932)	85.95^a^	13,331,7659^a^(919)	79.79^a^	15,176,3196(1086)	76.00^a^	15,592,9590^a^(1078)	75.72^a^
2	37	9,514^c^(256)	0.01^b^	53,324^c^(958)	0.04^b^	106,343^c^(1507)	0.07^c^	510,618^c^(4368)	0.32^d^	2,020,695^bc^(17107)	1.21^c^	4,938,362(41844)	2.47^c^	61,44,112^b^(53428)	2.98^c^
3	472	622,510^b^(334)	0.55^b^	3,508,974^b^(1237)	2.76^b^	7,745,821^b^(2067)	5.39^b^	14,258,079^b^(2964)	9.06^b^	19,506,141^b^(3504)	11.67^b^	27,747,408(5022)	13.90^b^	27,881,597^b^(5159)	13.54^b^
4	441	76^c^(1)	0.00^b^	134,185^c^(204)	0.10^b^	2,135,892^b^(1006)	1.49^bc^	7,213,948^b^(1748)	4.58^c^	11,880,724^b^(2370)	7.11^b^	14,225,586(3220)	7.12^b^	14,594,619^b^(3556)	7.09^c^
5	22	123,352^bc^(3846)	0.11^b^	102,609^c^(3195)	0.08^b^	124,963^c^(3616)	0.09^c^	131,216^c^(2937)	0.08^d^	365,547^c^(5487)	0.22^c^	1,016,667(16864)	0.51^c^	1,389,080^b^(25859)	0.68^d^

Means in the same column with different letters indicate a difference according to the Tukey test (P<0.05).

**Table 4:** Cluster of Brazilian municipalities according to the acceleration of growth in the number of cattle (Standard error) and the percentage of the total herd over the 5-year period.

Cluster	Number of municipalities	Cluster totals													
		77–81		82–86		87–91		92–96		97–01		02–06		07–11	
		N° head	%	N° head	%	N° head	%	N° head	%	N° head	%	N° head	%	N° head	%
1	416	475,276^b^(362)	0.42	2,136,630^b^(1,000)	1.68	4,902,490^b^(1,627)	3.41	8,995,882^b^(2,242)	5.71	13,554,254^b^(2,766)	8.11	23,221,157^b^(4,572)	11.64	26,225,864^b^(5,249)	12.75
2	48	9,513^c^(197)	0.01	231,130^c^(3,643)	0.18	359,335^c^(5,313)	0.25	621,111^c^(6,783)	0.39	1,651,891^c^(12,985)	0.99	3,943,384^c^(33,012)	1.98	4,687,262^c^(42,007)	2.28
3	689	4,665^c^(6)	0.00	422,420^c^(242)	0.33	3,638,908^b^(900)	2.53	11,707,855^b^(1629)	7.44	18,046,189^b^(1,849)	10.80	22,352,493^b^(2,452)	11.20	22,956,642^b^(2,558)	11.16
4	34	171,927^b^(2,541)	0.15	166,131^c^(2,172)	0.13	200,419^c^(2,434)	0.14	303,915^c^(3,544)	0.19	641,732^c^(7,961)	0.38	1,136,294^c^(12,952)	0.57	1,229,481^c^(14,206)	0.60
5	4364	112,173,230^a^(816)	99.41	124,238,215^a^(870)	97.68	134,642,308^a^(932)	93.67	135,795,385^a^(986)	86.26	133,196,461^a^(990)	79.72	148,910,406^a^(1,171)	74.62	150,607,922^a^(1,151)	73.21
	Total	112,834,613		127,194,528		143,743,461		157,424,150		167,090,528		199,563,736		205,707,173	

Means in the same column with different letters indicate a difference according to the Tukey test (P<0.05).

**Fig 5:**
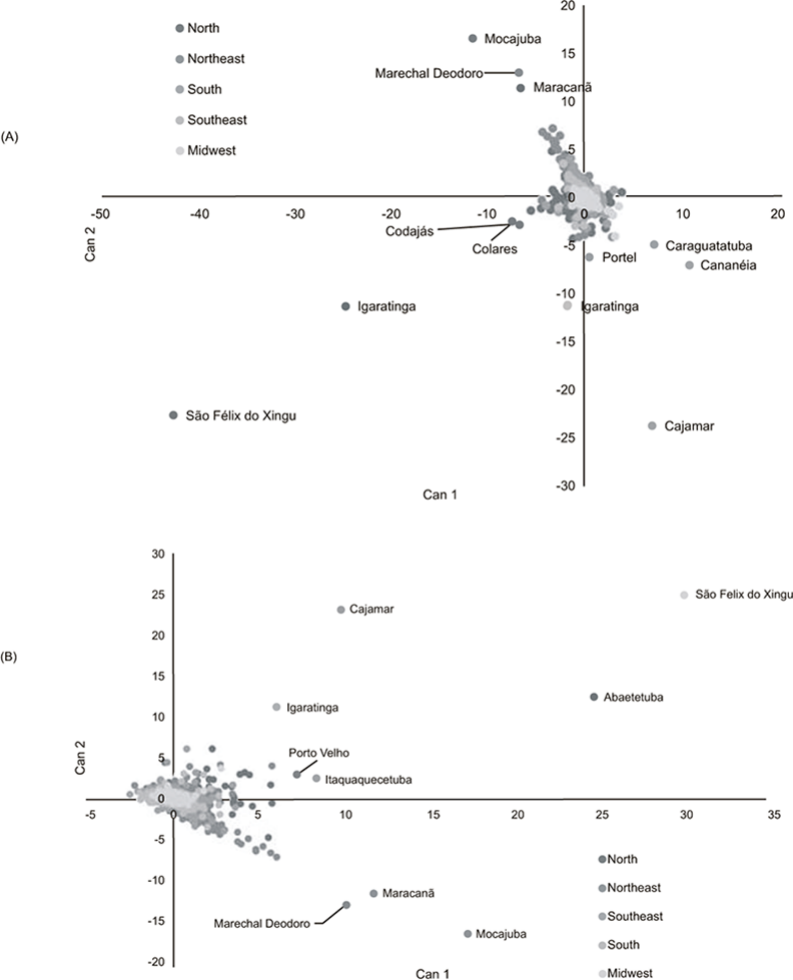
Canonical analysis of relative production) in cattle production in Brazil (N–North, NE–Northeast, SE–Southeast, S–South, and MW–Midwest). (Can 1 and Can 2 are the first tow canonical scores for each municipality). Each point on the graph represents a municipality, with those that are named showing highest discriminatory values.

**Fig 6:**
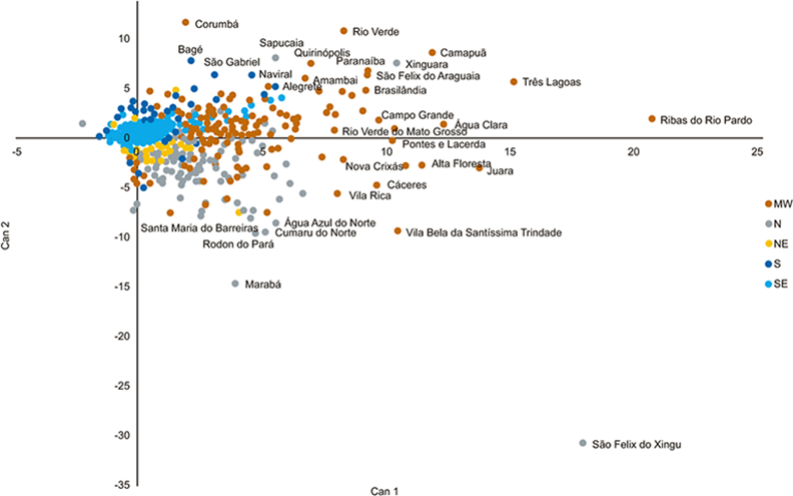
Canonical analysis of acceleration in cattle production in Brazil (N–North, NE–Northeast, SE–Southeast, S–South, and MW–Midwest). (Can 1 and Can 2 are the first tow canonical scores for each municipality). Each point on the graph represents a municipality, with those that are named showing highest discriminatory values.

**Fig 7:**
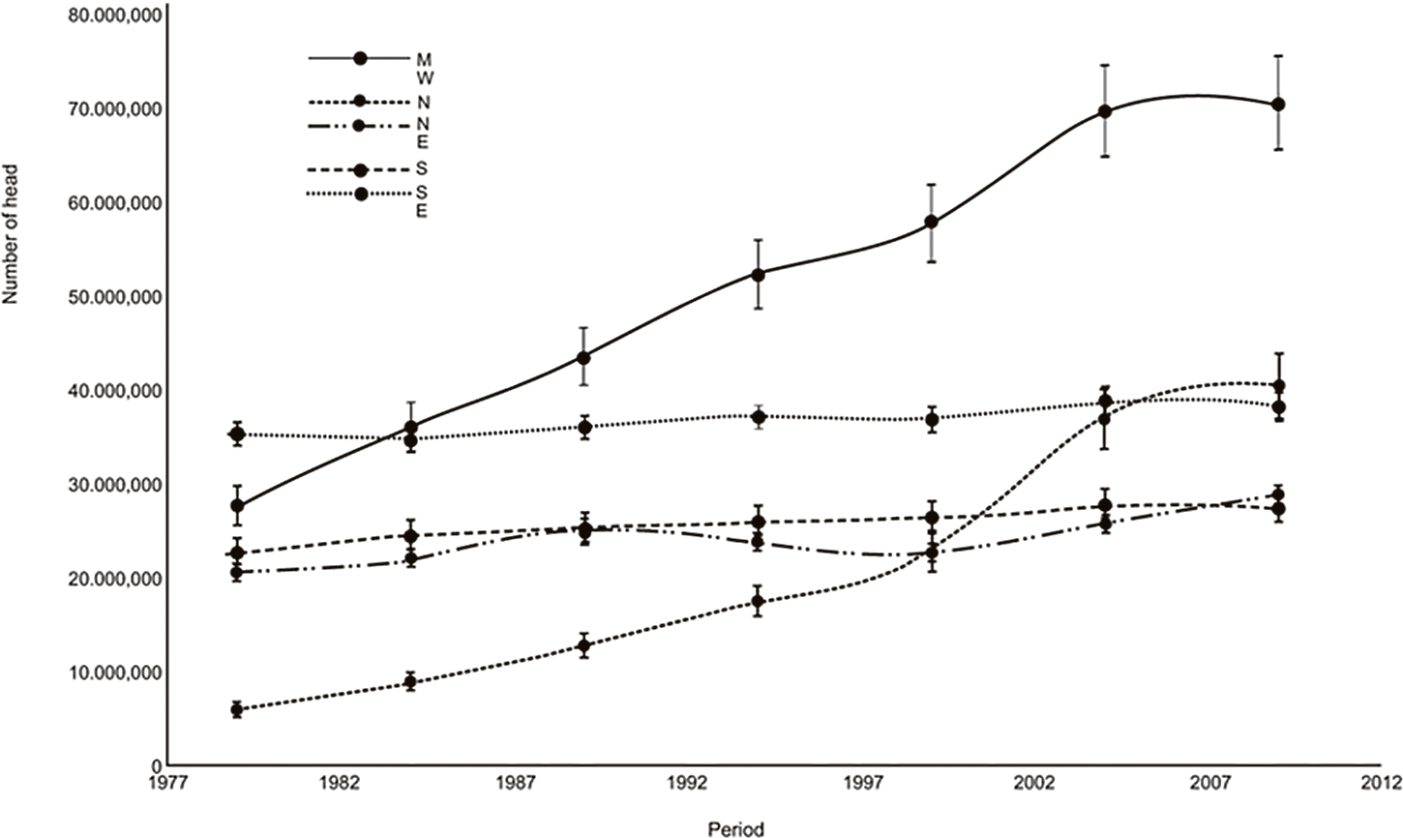
Total cattle numbers (periods of 5 years) in cattle production in Brazil (N–North, NE–Northeast, SE–Southeast, S–South, and MW–Midwest).

The canonical score plot of the first two canonical functions (Can1 and Can2) for each municipality are plotted in Figs 5–7. These reflect the highest variance in the discriminant model and provide a summary of the separation of the municipalities. Both increase and relative increases as well as acceleration of production are observed in the Midwest, Southeast and Northern regions, with only a few municipalities in each of these regions are responsible for these changes. These municipalities showed both marked growth and retraction in given periods. For example São Félix do Xingu in the Northern State of Pará went from less than 10,000 head to more than 1.9 million in the period studied, while Ribas do Rio Pardo, Corumbá, Juara and Cáceres, all in the Midwest, also saw significant increases in cattle numbers. Municipalities in the South (Alegrete, Bagé, São Gabriel), although showing reasonably high numbers of animals (approx 0.5 million), did not show significant changes in these numbers in the period shown, which may reflect the small variation in the herd of this region.

## Discussion

The causes of the geographical dynamics of the Brazilian cattle herd were originally associated with the need to meet the internal demand for beef, particularly in the 1970s and 1980s. The needs to expand the agricultural frontier in Brazil, the entry of foreign abattoir industries and the opening of markets to export processed beef to the United States have been motivated by the livestock sector and their migration to land areas at lower costs. The existence of a secure market stimulated investments in animal production and expansion to areas that were further from production centers. In this period, the driver was the existence of cheap land, which facilitated the increase in activity [8]. Subsequently, at the end of the 1990s, there was a greater increase in productivity rather than herd numbers, including a reduction in the area occupied by the activity.

Moreover, the need for Brazilian cattle growth in this decade was approximately 20%, however, the increase in productivity (weight kg/head/year) was around 30%, demonstrating a considerable gain in efficiency.

The adoption of technologies changed beef cattle production and productivity, particularly by increasing the weaning rate and weaning weight and reducing the age at slaughter and first mating. Between the years 2000–2010, weaning rate increased from 57 to 68%, with slaughter at 33 months of age, while weaning weight increased from 167 kg to 190 kg, and the age at first breeding of heifers went from 36 to 30 months. Technological developments in beef cattle production enabled Brazil to achieve an outstanding position in the international beef market. Between 1960 and 2010, the Brazilian cattle herd increased by 251%, and the stocking rate evolved from 0.47 head/hectare to 1.2 head/ha with an offtake rate of approximately 20% [9]. During this decade, the high prices of agricultural commodities, environmental restrictions and the high price of land in the Midwest region of Brazil, produced a new wave of cattle migration toward to the North, constituting 22% of the total effective of the country.

Intensification has been suggested as the means for the cattle industry [10] to reduce pressure on forest margins and free-up land for soybean or sugarcane production. The sugarcane expansion resulted in a significant reduction of pastures and number of cattle and higher economic growth compared to neighboring areas [11]. However, it could not be established to what extent the discontinuation of cattle production induced the expansion of pastures in other areas, thereby potentially resulting in indirect deforestation. However, these results indicate that the potential migration of cattle production reached further than the neighboring expansion regions. Therefore, a positive correlation between pasture, deforestation and cattle growth rate had existed in these Amazonian regions.

This is also the mainstay of Brazil's plan for the mitigation of greenhouse gas emissions [3], the environmental effect of animal production was crucial in the promotion of sustainability of agriculture production [12]. In the livestock sector, the animal productivity is related to food intake and weight gain efficiency, thereby generating methane emissions. Thus, the low beef cattle productivity index becomes the big problem of emission of greenhouse gases. This can be exacerbated by increased temperature in the region that can reduce up to 25% carrying capacity of pastures. All of these factors reinforce the need for seeking more efficient systems of land use with constant monitoring of its dynamics. Furthermore, the major drivers of sustainability in agriculture were the demands of the food market.

The original mid-point of cattle production was the so-called “Triângulo Mineiro or Mineiro Triangle” in the Minas Gerais State, which was the main region of cattle production in Brazil up to the turn of the millennium in terms of the number of animals as well as the history and culture of cattle ranchers. The Mato Grosso and Goias States constitute as a physiographic continuation of the north and northwest of the state of Minas Gerais, with no physical barriers that facilitated the displaced of cattle to similar geographical environment areas. In this region, the main zebu breeding centers are localized on the limits of the Midwest, where there is cheap land and perspectives for the expansion of zebu breeds, particularly the Nelore breed. The migration in cattle production was different from the migration in sheep production in Brazil during the same period [13], which followed a steady Northeastern route but has stabilized in recent years. This reflects the differences in production aims and market opportunities in the two production chains.

It is estimated that there is a decrease in beef cattle productivity due to the increase in air temperature and vulnerability of pasture capacity in the cerrado (savannah) Midwest, north and northeast regions of Brazil [14]. This may explain some of the results found in the present study. The increase in cattle production and expansion of pastures for cattle ranching in the North is accompanied by deforestation [15]. Recently, soy production has also moved into the Amazonian forest [16]. This occurred despite allegations of a widespread marketplace transition within the beef and soy industries, the main drivers of deforestation, to exclude Amazon deforesters from their supply chains [17]. Nevertheless, the expansion through the incorporation of forest areas, particularly in the northern regions, meets with barriers that did not occur in the past. Questions related to the new Brazilian Forest code, the lack of logistics (slaughterhouses, roads) for a more intensive production, as well as the difficulty in obtaining credit for investments in the region, caused serious restrictions for the accelerated migration of cattle to this region. This was reflected in a slow movement of cattle production into new regions (Figs 2 and 3) and the need to increase productivity. When examining the aptitude of the cattle herd for the region (beef and milk), 90% of the herd in the north and Midwest was for beef production. With better lands and climatic conditions in the south and southwest, milk production systems can account for up to 30% of all cattle production systems [18]. On the other hand, although do not occur growth in the North region, with the current flock and the structure of agro-industrial complex linked to the beef chain, there will be a demand for increased production in the livestock sector, which can be achieved by increasing productivity. It is noteworthy that this region weaning rate and head for productivity are 43 and 37% lower than the average of the Midwest, respectively.

Production does not mean competitiveness, and studies have shown a lack of competitiveness within Brazilian beef production [19, 20]. The total productivity factor show that technological growth in the agribusiness sector in Brazil was 4.5% per year, particularly in the southern region [21]. Nevertheless, productivity grew by only 2.6%. Thus, there was a gap between technological innovation and productivity. Restrictions to growth are due to the lack of credit, public policies directed to education, infrastructure and logistics. This may explain the deceleration in cattle production in regions that have problems with slaughterhouse logistics, such as transport and production flow, but may also be due to an increased productivity in the region, the production systems in the Amazon region still present low technology use [22].

However, it must be considered that the Brazilian cattle industry still has its production base in volume and scale in the Midwest and northern regions, as the price of land and the conditions of the biome limit more rapid expansion of agricultural crops. However, with the technological improvements in the agricultural sector and soils that are currently of limited use for farming should, in the future, present economic viability and expel livestock to new frontiers or would change the existing production systems for a more intensive processes by the semi-confinement and confinement systems which may maintain the current herd situation. This explains the movement of the cattle herd to the northern states of Brazil, as the land that had been previously occupied by livestock now produces soybeans and corn. Thus, production systems previously based on the complete cycle now lack conditions for fattening cattle, due to limitations of the pasture, a reduction in available physical space and logistics for the new region. However, this can affect the price of land for cattle ranching in Brazil, which increases in the same proportion as lands for agriculture.

The pro-intensification policies such as credit provision for recover degraded pastures and improved pasture management and investment in more intensive production systems must be accompanied by an implementation and enforcement of such policies [3]. These should alter the incentives to clear forest for pasture, discourage land speculation, and increase the accountability for land management practices if intensification of the cattle sector is to avoid new deforestation and displace production from low-yield, extensive cattle production systems in frontier regions of the Brazilian Amazon [1,23, 24].

**Fig 8:**
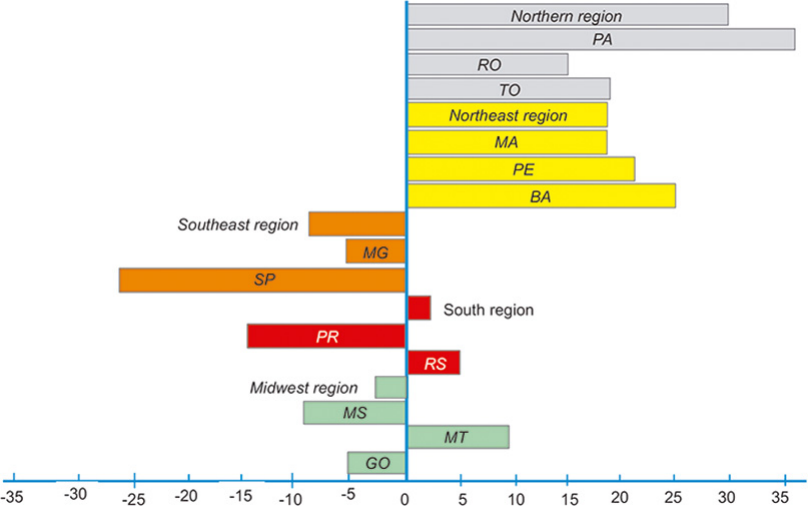
Variation in the rate of growth (%) of the cattle herd from 2005–2012 by region in Brazil and their main states (PA-Para, RO-Roraima, TO-Tocantins, MA-Maranhão, PE-Pernambuco, BA-Bahia, MG-Minas Gerais, SP-São Paulo, PR-Parana, RS-Rio Grande do Sul, MS-Mato Grosso, MT-Mato Grosso, and GO-Goiás.

Further analysis showed that during the period of greatest participation of Brazil in the international beef market and the valorization of agricultural produce (2005–2012), there was a relative commodity growth of 6%. However, this growth was higher in regions where land prices were lower (North and Northeast) (Fig 8). In these regions, there was an incentive to rear beef cattle, particularly for the installation of large meat processors and the implementation of large projects producing meat for export. However, due to environmental issues and port logistics and infrastructure, these regions failed to facilitate the sale of meat abroad. As an economic alternative, the state of Pará, the main producing state of these two regions, redirected its strategy for the export of live animals to countries such as Venezuela and Arab nations. Scenario studies have showed that Pará, 4th Brazilian herd, would have a low growth over the next 10 years, resulting in a stagnation of the regional herd. On the other hand, would improve productivity, especially in fattening due to adequacy of ranchers environmental legislation that enables the properties to provide animals for slaughter. Regardless of the slowing growth and significant reduction in herd numbers, the states of São Paulo, Goiás, Minas Gerais, Mato Grosso do Sul and Mato Grosso still represent the majority of beef exports in Brazil. These states are where the main structure for export of slaughtered animals is located, and they are also the main centers for completing feed-lots, which explains the fact that even with a reduction of the herd, they remain the leading exporters of meat.

Moving beef cattle production from one region to another requires skills and expertise as it passes through a redefinition of farming objectives, as well as strategies and processes to achieve better results. Nevertheless, there is always a risk that it will not work. Thus, there is a need to know the main vectors that will be employed in this migration. They may relate to the technological or organizational aspects of the main processes involved in the production cycle. This demonstrates that the change in land use for livestock immediately repositions other agricultural activities. In these areas, the farmer either sells land or remains with a reduction in herd size or may enhance productivity. Moreover, the emergence of integrated production systems called Crop-Livestock Integration and Crop-Livestock-Forest Integration can create new conditions for ranchers to avoid the migratory cycles for livestock production. These systems are based on sharing and maximizing the use of resources and the synergism between them, ensuring environmental balance and reducing trading risks by diversifying farm activities model. In addition, there might be a stabilization of farmers in their regions to acquire efficiency and sustainability.

## Conclusions

The acceleration of the cattle herd growth in Brazil has been increasing from 1977 to 2011. Thus, agricultural productivity must keep pushing cattle ranching to occupy new frontiers. However, the opening of new areas for livestock requires infrastructure, logistics, availability of credit/loans, public policies and adaptation to environmental issues that practically restrict this migratory phenomenon. These results confirm the reduction forecast in livestock productivity in the Cerrado, Midwest, North and Northeast due to the increase in air temperature and vulnerability of pastures.

Thus, it is clear that the expansion of the Brazilian herd is through migration, and if it finds no geoeconomical support, it should stabilize or even undergo a slight decrease as shown by the relative stagnation of this migration in recent years (Fig 1). In addition, there are possibilities of productivity gains in the beef cattle sector because there are still regions with low efficiency indicators. Therefore, the beef chain will be conducted by the market obligations. Thus, future studies should incorporate variables related to the expansion of crops, income from plant products, average area of farms, different specializations in cow-calf, growth and completion of production systems and density of cattle in different regions of Brazil.
